# A Multicentre, Randomised, Double Blind, Parallel Design, Placebo Controlled Study to Evaluate the Efficacy and Safety of Uthever (NMN Supplement), an Orally Administered Supplementation in Middle Aged and Older Adults

**DOI:** 10.3389/fragi.2022.851698

**Published:** 2022-05-05

**Authors:** Hao Huang

**Affiliations:** Effepharm (Shanghai) Co., Ltd., Shanghai, China

**Keywords:** NADH, middle aged, anti-ageing, Uthever, randomised controlled trial, HOMA, SF-36, NMN

## Abstract

**Objective:** The purpose of the study was to evaluate the anti-aging effect of NMN and its safety in a double-blind, parallel, randomised controlled clinical trial.

**Methods:** The study was carried out on 66 healthy subjects between the ages of 40 and65 years, instructed to take two capsules (each containing 150 mg. of NMN or starch powder) once a day after breakfast for 60 days.

**Results:** At day 30, NAD^+^/NADH levels in the serum showed a noteworthy increase, i.e., by 11.3%, whereas the placebo group had shown no change at all. At the end of the study, i.e., day 60, the NAD^+^/NADH levels were increased further by 38% compared to baseline, against a mere 14.3% in the placebo group. In the case of SF 36, at day 60, the Uthever group showed a rise of 6.5%, whereas the placebo group was merely raised by 3.4%. At the end of the study, the mean HOMA IR Index showed a rise of 0.6% among the Uthever group and 30.6% among the Placebo group from baseline.

**Conclusion:** The rise in the levels of NAD^+^/NADH at day 30 and day 60 illustrated the potential of Uthever to raise the levels of NAD^+^ in the cells, which is linked to higher energy levels and an anti-aging effect. Increased sensitivity to insulin has also been linked to anti-aging. There was no noteworthy change in HOMA score, in the Uthever group whereas there was a noteworthy rise in the placebo group, demonstrating the anti-aging effect of Uthever as in its absence, the parameters worsened.

**Clinical Trial Registration:** (clinicaltrials.gov), identifier (NCT04228640 NMN).

## Introduction

Advancements in medical sciences have resulted in an overall increase in the lifespan of the human world. This has led to an incessant burden on governmental finances in order to make aging healthy, sparking the interest of scientists worldwide in exploring anti-aging compounds. Several pharmaceutical drugs such as metformin, rapamycin, and SIRT1 activators are available in the market and are being explored as anti-aging drugs through clinical trials (Barzilai 2017; Campisi et al., 2019, NCT04488601). Researchers are in search of some endogenous compounds that might have the potential to achieve healthy and productive lives even at old age. Nicotinamide mononucleotide (NMN) is one such endogenous molecule. Nicotinamide mononucleotide (NMN) is a bioactive nucleotide naturally synthetised from a phosphate group and nicotinamide riboside by the enzyme NAMPT (Bieganowski and Brenner, 2004). NMN is an intermediate molecule in nicotinamide adenine dinucleotide (NAD^+^) biosynthesis (Hsu et al., 2010), which acts as an important substrate for various enzymes that help in the enzymatic conversion to NAD^+^ in humans (Berger et al., 2005). NAD^+^ is an essential cofactor in all living cells involved in fundamental biological processes. Depletion in the levels of NAD^+^ is associated with traits of aging, many age-related diseases like cancer, metabolic disorders, and neurological disorders (Aman et al., 2018).

It is known that aging is characterised by down-regulated energy production by mitochondria, owing to the depletion of NAD^+^ in multiple organs like the pancreas, skeletal muscle, liver, skin, adipose tissue, and brain (Mills et al., 2016; Mouchiroud et al., 2013; Kincaid and Bossy-Wetzel, 2013). A study has shown that an increased level of NAD^+^ consuming enzymes, e.g., NAD^+^ dependent acetylase (Sirtuins), poly ADP-ribose polymerase (PARP), and NADase (CD38), contribute to the decline of NAD^+^ with age (Camecho-Pereira et al., 2016). Along with these there are other changes occurring like DNA damage, cognitive impairment, sirtuin gene inactivation which can be retreated by NAD^+^. It is believed that the administration of NMN can compensate for the deficiency of NAD^+^ caused by these NAD^+^ consuming enzymes. An *in vitro* study (Berger et al., 2005) found that three isoforms of human nicotinamide mononucleotide adenylyl-transferase (NMNAT) catalyse the conversion of NMN to NAD^+^ by catalysing the reversible reaction NMN + ATP ⇌ NAD^+^ + PPi.

In addition to this, NMN and NR have fewer adverse effects than other NAD^+^ precursors (Cantó et al., 2012; Mackay et al., 2012).

Various preclinical trials demonstrated the diversified pharmacological activities of NMN in cardiac and cerebral ischemia, Alzheimer’s disease, diet-and age-induced type 2 diabetes, and obesity, all of which are linked to the deficiency of NAD^+^ (Yoshino et al., 2011; Yamamoto et al., 2014; Long et al., 2015; Fang et al., 2017). Recent evidence is shaping a picture where low caloric regimes and exercise may improve healthy senescence, and several pharmacological strategies have been suggested to counteract aging. The most effective interventions proposed to date converge on only a few cellular processes, in particular nutrient signalling, mitochondrial efficiency, proteostasis, and autophagy (de Cabo et al., 2014).

Promising results in pre-clinical trials led to the evaluation of NMN in human subjects. A single-arm non-randomised intervention was conducted by single oral administration of 100, 250, and 500 mg of NMN in 10 healthy Japanese men and found NMN safe for human use (Junichiro et al., 2020). It has also been explored in obese postmenopausal women with pre-diabetes for 10 weeks to determine its effect on muscle insulin sensitivity (Yoshino et al., 2021). NMN is currently being evaluated for effects on cardiometabolic function, for efficacy in hypertensive patients, and as an anti-aging supplement at doses ranging from 200 to 500 mg/day (US trial registry no. NCT03151239, NCT03151239, NCT04910061, NCT04862338, NCT04823260, NCT04571008, NCT04903210, and NCT04664361) for a duration of 29 days–16 weeks.

There is limited data available for the efficacy of NMN as an anti-aging supplement. The present study design is based on available pre-clinical data for NMN as a supplementation. The purpose of this study was to evaluate the safety and efficacy of Uthever, a nicotinamide mononucleotide (NMN) supplement, in the stimulation of NAD^+^ metabolism in middle-aged and older adults, thereby assessing its anti-ageing effect in 66 adult and elderly subjects.

## Materials and Methods

### Study Population

**FIGURE 1:**
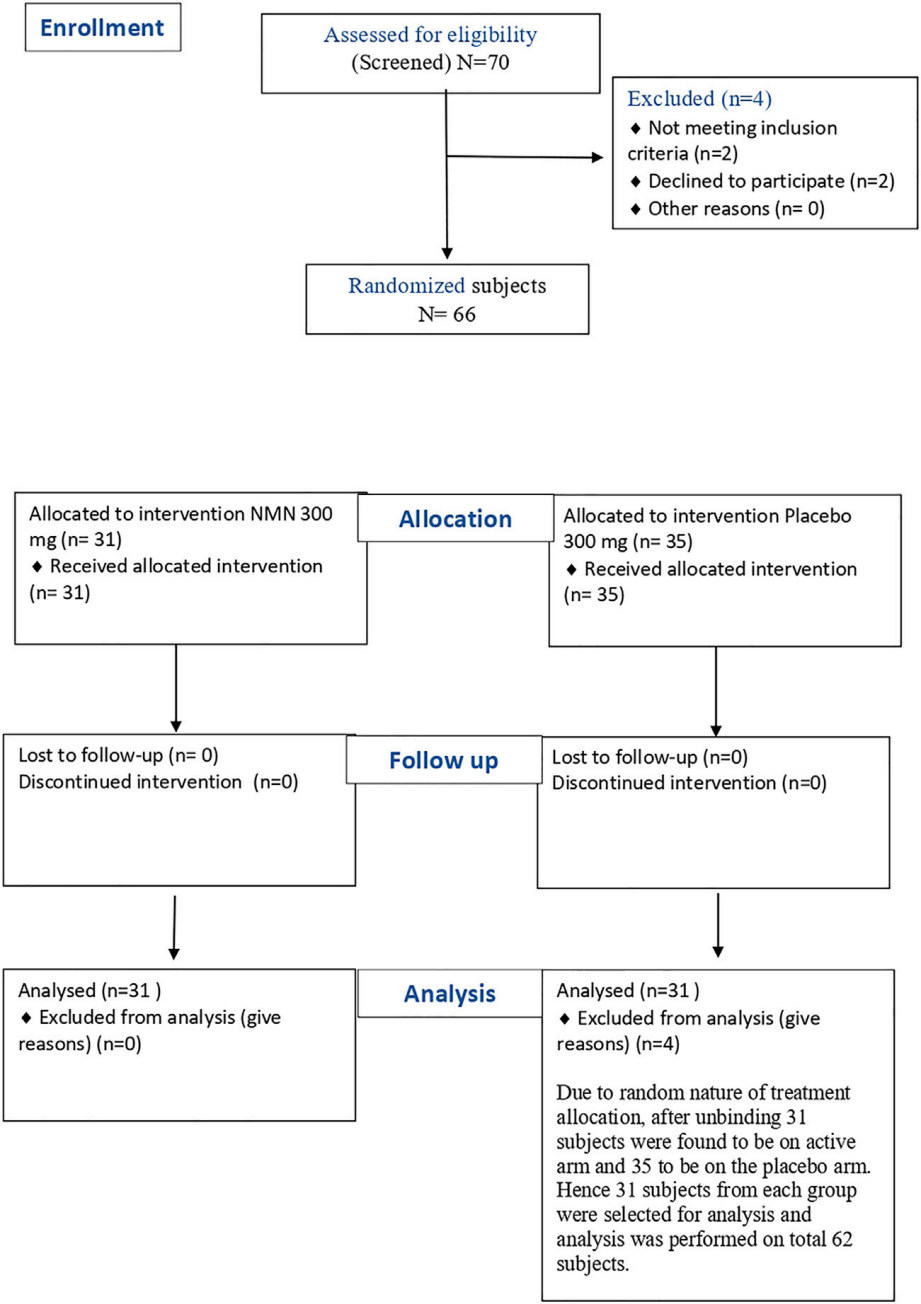
Flow diagram for subject distribution.

The study was carried out on healthy subjects between the ages of 40–65 years, and the BMI (Body Mass Index) was between 18.5 and 35 kg/m^2^. The subjects were determined to be healthy by assessing vital signs, physical examination, and medical history, including any concomitant medications and several laboratory tests (haematology, clinical chemistry, and urinalysis), electrocardiograph (ECG), and X-Ray during screening. The study did not include people with atherosclerotic disease and/or cardiopulmonary diseases, history of drug abuse or alcohol abuse, unstable mental illness that could impact a participant’s ability to comply with the study, those that used statins, or pregnant or lactating women. The screening was performed fourdays before dosing. A total of 70 potential subjects were screened out, of which 66 were found to be eligible based on all the inclusion and exclusion criteria. These 66 subjects were randomised to either the NMN arm or the placebo arm according to the randomisation scheme in a 1:1 ratio. After unblinding, 31 subjects were found to be in the active arm and 35 to be in the placebo arm. Hence, 31 subjects from each group were selected randomly to avoid any skewing of data in the analysis. The analysis was performed on a total of 62 subjects Figure 1.

### Study Design

This study was conducted as a randomised, multi-centre, double-blind, placebo-controlled, parallel-design clinical trial. It was performed at Swasthya clinic & research centre and Nirmaya hospital in Pune, India. Prior to study initiation, the Royal Ethics Committee, Pune, India had reviewed the clinical study protocol, informed consent form, and corresponding translations of ICF (Hindi and Marathi) and had accorded its approval in the meeting held onJanuary 6, 2020. The independent ethics committee was formed as per the new drugs and clinical trials rules, 2019 and is duly registered with the Drugs Controller General, India *via* number - ECIV45/Indt/MII/2013/RR-19. This study was carried out according to good clinical practice (GCP) and the principles of the Declaration of Helsinki. All subjects were informed of the objective, content, and risks of the study and voluntarily signed the written informed consent form before enrolment.

### Efficacy and Safety Evaluation

All enrolled subjects were instructed to take two capsules (each containing 150 mg. NMN/starch powder) of either Uthever (NMN supplement) or placebo once a day after breakfast for 60 days. To evaluate the efficacy of NMN (Uthever) in stimulating NAD^+^ metabolism and its effect on middle-aged and older subjects, several parameters like blood cellular NAD^+^/NADH concentration in serum, 6 min walking endurance test, systolic and diastolic blood pressure, pulse pressure, and the SF-36 questionnaire were assessed as primary endpoints in the study. HOMA (homeostatic model assessment) was also assessed as an exploratory endpoint. To test the safety of NMN at dose of 300 mg for 60 days the safety laboratory tests and organ function tests were kept. These safety assessments were performed at baseline and at the end of the study visit. Hence, any significant side effects would be seen through the de-ranged laboratory values and organ function tests. As a result, any clinically significant worsening of the parameters compared to no treatment (as shown at baseline) would have indicated that the active group has a negative impact and has safety concerns.

The NAD^+^/NADH concentration in serum was tested by a colorimetric quantification kit from MyBioSource (CA, USA). The assay is based on an enzymatic cycling reaction in which NAD^+^ is reduced to NADH. NADH reacts with a colorimetric probe to produce a colored product that is measured at 450 nm. The intensity of the product color is proportional to the NAD^+^ and NADH within a sample. A simple acid or base treatment differentiated NADH from NAD^+^ within a sample. Samples and standards were incubated for 1–4 h and then read with a manual ELISA reader (PR4100) from BioRad Laboratories (CA, USA). Samples were compared to a known concentration of NAD^+^ standard within the 96-well micro titre plate format.

The HOMA IR Index was calculated using the HOMA2 IR calculator by the University of Oxford Diabetes Trial Unit. The calculator uses fasting blood glucose and fasting insulin levels in serum. Blood samples from subjects were collected for HOMA after overnight fasting (at least 8 hof fasting).

To assess the improvement in physical performance and energy, a 6 min walk test was conducted where the subject had to walk on a 50-foot indoor course and the distance walked by the subject in 6 mins was measured (Enright, 2003).

To assess improvement in general well-being of the subject, the SF-36 questionnaire was used, in which the subject had to answer 36 questions related to energy, emotions, social activities, and physical health of subjects (Ware et al., 1992).

### Statistical Analysis

The primary objectives were evaluated at day 30 and day 60 after treatment initiation. Data collected from both the sites, i.e., Swasthya clinic & research centre and Nirmaya hospital, was used for analysis. All the efficacy analysis was performed on a per-protocol subset (PP), i.e., subjects without any major protocol violations would be included in the per-protocol population, including those subjects who have completed all the visits and who did not take any prohibited medications during the study period. The study did not have any violations for any of the subjects. None of the subjects were excluded from the efficacy analysis because of any adverse event or any violation. A comparison of the active and placebo treatment arms was undertaken, with changes in results from baseline to the end of the study being summarised by treatment.

The categorical variables were expressed as numbers and percentages. All the primary efficacy parameters were summarised using statistical methods appropriate for each type of data set and were tabulated for each time point for the parameters reported. The primary efficacy endpoints were analysed using the student “t” test. For a few variables, a non-parametric approach was used, which employed the Wilcoxon Sign Rank Test and Mann Whitney *U* Test for endpoints like SF-36, mean HOMA IR index, and mean serum insulin.

## Results

### Demographics of the Subject Population

The subject demographic and baseline characteristics were very similar among the two treatment groups; the overall mean age was approximately 47 years and the subjects were predominantly female (Table 1).

**TABLE 1:** Subject demographics summary.

Parameters	Uthever (*N* = 31)	Placebo (*N* = 31)	*p* Values
^a^Age (years)			0.74
Mean	47.76	47.21	
SD	06.60	06.55	
Range	40.00–64.00 years	40.00–64.00 years	
^a^Weight (kg)			0.39
Mean	62.10	60.83	
SD	06.14	05.61	
Range	50.00–72.00 kg	53.00–76.00 kg	
^a^Height (cm)			0.81
Mean	156.73	156.97	
SD	04.03	04.18	
Range	149.00–162.00 cm	146.00–164.00 cm	
^a^BMI (kg/m^2^)			0.37
Mean	25.26	24.72	
SD	02.34	02.40	
Range	21.60–29.20 kg/m^2^	20.10–30.30 kg/m^2^	
^b^Sex			0.60
Male	13 (41.9)	15 (48.4)	
Female	18 (58.1)	16 (51.6)	

a By Student t test.

b By Chi-Square Test *p* > 0.05 was considered not significant.

### Blood Cellular NAD^+^/NADH

The primary efficacy parameter, NAD^+^/NADH levels in the serum, had increased by 11.3% in the active group (Uthever group) at day 30, whereas no change was observed in the placebo group at all. At the end of the study (day 60), the NAD^+^/NADH levels were increased further by 38% from baseline in the Uthever group, compared to a mere 14.3% rise in the placebo group (Table 2). The increase in the placebo group may be attributed to the placebo effect in this study. Although the difference between the active and placebo groups is not statistically significant, the results indicate that Uthever does increase the NAD^+^ levels in the serum after 2 months of duration as well.

**TABLE 2:** Comparison of changes in mean blood cellular NAD^+^/NADH between the groups.

Duration (Days)	Mean blood cellular NAD^+^/NADH (pmol/ml) ( $\bar{Χ}$ ± SD)		*p* value
	Uthever (*N* = 31)	Placebo (*N* = 31)	
Baseline	6.57 ± 4.36	7.12 ± 4.56	0.62
30	7.31 ± 6.82	7.12 ± 5.66	—
60	9.07 ± 5.65	8.14 ± 4.86	—
Mean diff (Baseline—Day 30) (*p* value)	0.73 ± 8.38 (0.63)	0.00 ± 6.61 (1.0)	0.70
Mean diff (Baseline—Day 60) (*p* value)	2.50 ± 8.21 (0.10)	1.01 ± 5.35 (0.30)	0.40

By Student “t” Test *p* > 0.05 was considered not significant.

### 6 Minute Walking Endurance Test

The walking endurance increased by 4.3% in the Uthever group and 3.9% in the placebo group on day 30 of the treatment. So no effective difference was seen on day 30 of the treatment for walking endurance. When the same treatment was continued up to day 60, the Uthever group showed a rise of 6.5% where as for the placebo group, it remained the same, i.e., 3.9% (Table 3). The findings were not found to be statistically significant.

**TABLE 3:** Comparison of changes in total distance covered in (6 Minute Walk Test) between the groups.

Duration (Days)	Mean total distance covered (kilometres) ( $\bar{Χ}$ ± SD)		*p* value
	Uthever (*N* = 31)	Placebo (*N* = 31)	
Baseline	0.46 ± 0.03	0.51 ± 0.24	0.25
30	0.48 ± 0.02	0.53 ± 0.26	—
60	0.49 ± 0.02	0.53 ± 0.27	—
Mean diff (Baseline–Day 30) (*p* value)	*0.02 ± 0.04 (0.01)	*0.02 ± 0.03 (0.01)	0.10
Mean diff (Baseline–Day 60) (*p* value)	*0.03 ± 0.04 (0.00)	*0.02 ± 0.05 (0.03)	0.38

By Student “t” Test *p* > 0.05 was considered not significant.

From this analysis, it was clear that the placebo effect was evident until day 30, but after that, the Uthever group showed further improvement in walking endurance, whereas the placebo group remained at the same level.

### SF-36

The SF 36 questionnaire demonstrates the wellbeing of the subject. the higher the score, the better the health of the subjects.

On day 30, in the Uthever group, the score was raised by 4.0%, whereas in the placebo group it was raised by 3.7%. So there is no meaningful difference in the score at day 30. At day 60, the Uthever group showed a rise of 6.5%, whereas the placebo group was merely raised by 3.4%. The difference in SF-36 scores between the active and placebo groups was not statistically significant, but the increase in scores in the Uthever group was almost double the increase seen in the placebo group.

### Pulse Pressure, Systolic and Diastolic Blood Pressure

Reduction towards normalisation of pulse pressure, systolic and diastolic blood pressure was considered as a parameter for evaluation of the anti-ageing effect of Uthever. At day 30, mean pulse pressures showed a fall of 2.8% among the Uthever group and 5.6% among the placebo group from baseline. The mean systolic blood pressure showed a fall of 0.3% among the Uthever group and 1.1% among the placebo group from baseline. The mean diastolic blood pressure showed a fall of 2.2% among the Uthever group and a rise of 0.2% among the placebo group from baseline (Tables 4, 5). The findings were not statistically significant.

**TABLE 4:** Comparison of changes in mean total score of SF-36 questionnaire between the groups.

Duration (Days)	Mean total score of SF-36 questionnaire ( $\bar{Χ}$ ± SD)		*p* value
	Uthever (*N* = 31)	Placebo (*N* = 31)	
Baseline	132.77 ± 8.91	129.64 ± 17.04	0.37
30	138.15 ± 7.45	134.52 ± 14.48	—
60	141.36 ± 8.62	134.04 ± 18.06	—
Mean diff (Baseline–Day 30) (*p* value)	*5.38 ± 08.94 (0.00)	*4.88 ± 07.83 (0.00)	0.82
Mean diff (Baseline–Day 60) (*p* value)	*8.59 ± 11.83 (0.00)	*4.40 ± 16.06 (0.13)	0.25

By Wilcoxon Sign Rank Test *p* > 0.05 was considered not significant.

By Mann Whitney *U* Test.

**TABLE 5:** Comparison of changes in mean pulse pressure, systolic and diastolic blood pressure between the groups.

Duration (Days)	Mean SBP (mmHg) ( $\bar{Χ}$ ± SD)		*p* value	Mean DBP (mmHg) ( $\bar{Χ}$ ± SD)		*p* value	Mean PP (mmHg) ( $\bar{Χ}$ ± SD)		*p* value
	Uthever (*N* = 31)	Placebo (*N* = 31)		Uthever (*N* = 31)	Placebo (*N* = 31)		Uthever (*N* = 31)	Placebo (*N* = 31)	
Baseline	124.26 ± 14.73	128.90 ± 15.84	0.23	78.65 ± 8.76	79.06 ± 7.46	0.84	49.42 ± 17.46	51.19 ± 12.38	0.64
30	123.84 ± 15.49	127.52 ± 11.65	—	76.94 ± 9.00	79.19 ± 6.90	—	48.03 ± 12.46	48.32 ± 10.93	—
60	124.19 ± 12.03	126.26 ± 13.51	—	76.81 ± 8.30	77.84 ± 9.01	—	49.23 ± 11.09	48.10 ± 12.02	—
Mean diff (Baseline –Day 30) (*p* value)	−0.42 ± 11.10 (0.83)	−1.39 ± 14.76 (0.60)	0.77	−1.71 ± 7.63 (0.22)	0.13 ± 8.09 (0.92)	0.36	−1.39 ± 18.37 (0.67)	−2.87 ± 13.75 (0.25)	0.72
Mean diff (Baseline –Day 60) (*p* value)	−0.06 ± 10.31 (0.97)	−2.65 ± 14.72 (0.32)	0.42	−1.84 ± 8.51 (0.23)	−1.23 ± 9.90 (0.49)	0.79	−0.19 ± 18.59 (0.95)	−3.10 ± 14.02 (0.22)	0.48

By Student “t” Test *p* > 0.05 was considered not significant.

### HOMA

To evaluate the exploratory anti-aging effect on insulin regulation towards normalisation, the HOMA IR index was assessed, along with fasting blood sugar and serum insulin (fasting). At the end of the study, the mean HOMA IR index showed a rise of 0.6% among the Uthever group and a rise of 30.6% among the placebo group from baseline. Mean Glucose (Sugar) fasting showed a fall of 4.0% among the Uthever group and rise of 6.5% among the placebo group from baseline. Mean Serum Insulin fasting showed a fall of 1.9% among the Uthever group whereas a rise of 26.2% among the placebo group from baseline (Tables 6–8). The difference in HOMA IR Index between the Uthever and the placebo groups was not found to be statistically significant.

**TABLE 6:** Comparison of changes in mean HOMA IR index between groups.

Duration (Days)	Mean HOMA IR index ( $\bar{Χ}$ ± SD)		*p* value
	Uthever (*N* = 31)	Placebo (*N* = 31)	
Baseline	1.78 ± 0.85	1.83 ± 1.15	0.85
60	1.79 ± 0.94	2.39 ± 2.08	—
Mean diff (Baseline –Day 60) (*p* value)	0.01 ± 0.90 (0.95)	0.55 ± 2.02 (0.14)	0.18

By Wilcoxon Sign Rank Test *p* > 0.05 was considered not significant.

By Mann Whitney Test.

**TABLE 7:** Comparison of changes in mean blood glucose (fasting) between groups.

Duration (Days)	Mean glucose (sugar) fasting (mg/dl) ( $\bar{Χ}$ ± SD)		*p* value
	Uthever (*N* = 31)	Placebo (*N* = 31)	
Baseline	95.33 ± 20.98	101.88 ± 27.04	0.29
60	91.53 ± 21.41	108.48 ± 47.12	—
Mean diff (Baseline –Day 60) (*p* value)	−3.80 ± 19.03 (0.27)	6.60 ± 42.22 (0.39)	0.21

By Student “t” Test *p* > 0.05 was considered not significant.

**TABLE 8:** Comparison of changes in mean serum insulin (fasting) between groups.

Duration (Days)	Mean serum insulin fasting (microU/ml) ( $\bar{Χ}$ ± SD)		*p* value
	Uthever (*N* = 31)	Placebo (*N* = 31)	
Baseline	14.33 ± 6.80	15.12 ± 9.25	0.70
60	14.06 ± 7.52	19.08 ± 18.09	—
Mean diff (Baseline –Day 60) (*p* value)	−0.26 ± 7.59 (0.85)	3.96 ± 17.37 (0.21)	0.22

By Wilcoxon sign rank test by Mann Whitney test.

*p* > 0.05 was considered not significant.

### Safety Analysis

Two patients had adverse events of dyslipidaemia, one of which was in the active group and the other in the placebo group. Both the subjects had mild dyslipidaemia, which was completely resolved by giving medication, and the causality was unlikely. The adverse events profile states that 3.2% of cases among both groups had adverse events, which was the same amongst both groups (Table 9). The severity of events in both cases was mild.

**TABLE 9:** Profile of adverse events.

Adverse events	Uthever (*N* = 31)		Placebo (*N* = 31)	
	No.	%	No.	%
Dyslipidemia	01	03.2	01	03.2
Total No. of Events	01	—	01	—
Total No. of subjects	01	03.2	01	03.2

By fisher exact test.

There was no clinically meaningful derangement (worsening) in safety laboratory tests in both groups, which indicates the safety of Uthever (NMN) supplement (Tables 10–13).

**TABLE 10:** Comparison of mean laboratory data (CBC, Hematology) between groups.

Laboratory tests	Duration (Days)	Mean ( $\bar{Χ}$ ± SD)		*p* value
		Uthever (*N* = 31)	Placebo (*N* = 31)	
Hemoglobin (g/dl)	Baseline	13.32 ± 1.65	13.28 ± 1.86	0.92
	60	13.05 ± 1.68	13.32 ± 1.97	—
	Mean diff (Baseline –Day 60) (*p* value)	−0.27 ± 0.66 (0.03)	0.04 ± 1.03 (0.83)	0.16
Hematocrit—PCV (%)Total	Baseline	40.78 ± 4.37	39.40 ± 8.05	0.40
	60	39.73 ± 4.36	40.85 ± 5.06	—
	Mean diff (Baseline –Day 60) (*p* value)	−1.05 ± 2.03 (0.00)	1.46 ± 7.26 (0.27)	0.06
Total Leukocyte Count-WBC Total Count (/cmm)	Baseline	6745.16 ± 1650.62	6612.90 ± 1289.64	0.72
	60	6296.77 ± 1813.01	6490.32 ± 1359.25	—
	Mean diff (Baseline –Day 60) (*p* value)	−448.39 ± 1662.50 (0.14)	−122.58 ± 899.89 (0.45)	0.34
Neutrophils (%)	Baseline	55.88 ± 7.87	53.09 ± 9.66	0.21
	60	56.41 ± 8.15	55.14 ± 9.45	—
	Mean diff (Baseline –Day 60) (*p* value)	0.53 ± 7.46 (0.69)	2.05 ± 6.86 (0.10)	0.40
Absolute Neutrophils (/cmm)	Baseline	3682.93 ± 1439.17	4185.51 ± 4267.12	0.53
	60	4698.02 ± 5771.45	3635.80 ± 1205.23	—
	Mean diff (Baseline –Day 60) (*p* value)	1015.09 ± 5672.56 (0.32)	−549.72 ± 4396.63 (0.49)	0.22
Lymphocytes (%)	Baseline	33.79 ± 6.12	35.36 ± 6.89	0.34
	60	34.61 ± 6.71	35.45 ± 9.51	—
	Mean diff (Baseline –Day 60) (*p* value)	0.82 ± 7.39 (0.54)	0.08 ± 6.70 (0.94)	0.68
Monocytes (%)	Baseline	5.82 ± 1.77	5.91 ± 1.93	0.84
	60	4.67 ± 1.49	4.87 ± 1.51	—
	Mean diff (Baseline –Day 60) (*p* value)	−1.15 ± 1.72 (0.00)	−1.03 ± 1.55 (0.00)	0.77
Basophils (%)	Baseline	0.00 ± 0.00	0.01 ± 0.03	0.06
	60	0.01 ± 0.02	0.01 ± 0.03	—
	Mean diff (Baseline –Day 60) (*p* value)	0.01 ± 0.02 (0.00)	0.00 ± 0.05 (1.0)	0.30
Eosinophils (%)	Baseline	4.27 ± 4.47	4.60 ± 2.94	0.73
	60	4.19 ± 3.43	4.53 ± 3.12	—
	Mean diff (Baseline –Day 60) (*p* value)	−0.09 ± 1.79 (0.78)	−0.07 ± 1.49 (0.79)	0.96
Platelet Count (/cmm)	Baseline	268141.94 ± 89517.44	276096.77 ± 75638.77	0.70
	60	272806.45 ± 81622.88	265764.52 ± 87540.73	—
	Mean diff (Baseline –Day 60) (*p* value)	4664.52 ± 47631.74 (0.58)	−10332.26 ± 58404.87 (0.33)	0.27
Red blood cell count (mil/cmm)	Baseline	4.66 ± 0.58	4.59 ± 0.57	0.63
	60	4.64 ± 0.61	4.58 ± 0.55	—
	Mean diff (Baseline –Day 60) (*p* value)	−0.02 ± 0.24 (0.64)	−0.00 ± 0.36 (1.0)	0.79
Prothrombin time (seconds)	Baseline	3.72 ± 5.22	4.89 ± 5.42	0.39
	60	3.82 ± 4.94	4.92 ± 6.43	0.39
	Mean diff (Baseline –Day 60) (*p* value)	0.10 ± 4.76 (0.90)	0.02 ± 5.51 (0.98)	0.95
Activated Partial Thromboplastin Time (seconds)	Baseline	29.61 ± 3.36	28.91 ± 3.01	0.39
	60	29.81 ± 3.64	30.22 ± 3.20	—
	Mean diff (Baseline –Day 60) (*p* value)	0.20 ± 3.55 (0.75)	1.32 ± 3.03 (0.02)	0.18

By Student “t” test *p* > 0.05 was considered not significant.

**TABLE 11:** Comparison of mean laboratory data (LFT) between groups.

Laboratory tests	Duration (Days)	Mean ( $\bar{Χ}$ ± SD)		*p* value
		Uthever (*N* = 31)	Placebo (*N* = 31)	
Total Bilirubin (mg/dl)	Baseline	0.62 ± 0.27	0.59 ± 0.27	0.66
	60	0.63 ± 0.27	0.60 ± 0.26	—
	Mean diff (Baseline –Day 60) (*p* value)	0.01 ± 0.18 (0.75)	0.01 ± 0.18 (1.0)	0.82
Aspartate aminotransferase (U/L)	Baseline	19.75 ± 5.14	19.62 ± 5.99	0.92
	60	18.31 ± 4.09	18.97 ± 4.11	—
	Mean diff (Baseline –Day 60) (*p* value)	−1.44 ± 4.79 (0.10)	−0.65 ± 5.09 (0.48)	0.53
Alanine aminotransferase (U/L)	Baseline	20.28 ± 9.55	18.75 ± 7.00	0.47
	60	17.20 ± 6.58	19.45 ± 8.77	—
	Mean diff (Baseline –Day 60) (*p* value)	−3.08 ± 8.33 (0.04)	0.70 ± 7.34 (0.59)	0.06
Alkaline phosphatase (U/L)	Baseline	75.50 ± 25.67	83.68 ± 35.94	0.30
	60	76.04 ± 25.93	85.58 ± 31.91	—
	Mean diff (Baseline –Day 60) (*p* value)	0.54 ± 13.78 (0.82)	1.90 ± 14.32 (0.46)	0.70

By Student “t” test *p* > 0.05 was considered not significant.

**TABLE 12:** Comparison of mean laboratory data (RFT) between groups.

Laboratory tests	Duration (Days)	Mean ( $\bar{Χ}$ ± SD)		*p* value
		Uthever (*N* = 31)	Placebo (*N* = 31)	
Glomerular Filtration Rate (ml/min/1.73sqm)	Baseline	94.26 ± 16.42	92.23 ± 21.25	0.67
	60	92.71 ± 16.56	90.52 ± 17.67	—
	Mean diff (Baseline –Day 60) (*p* value)	−1.55 ± 13.15 (0.51)	−1.72 ± 10.85 (0.38)	0.95
Urea (mg/dl)	Baseline	18.35 ± 4.63	20.52 ± 6.28	0.12
	60	17.79 ± 5.37	20.30 ± 7.06	—
	Mean diff (Baseline –Day 60) (*p* value)	−0.56 ± 5.87 (0.59)	−0.22 ± 5.24 (0.81)	0.81
Blood Urea Nitrogen (mg/dl)	Baseline	8.58 ± 2.16	9.59 ± 2.93	0.12
	60	8.37 ± 2.50	9.44 ± 3.34	—
	Mean diff (Baseline –Day 60) (*p* value)	−0.21 ± 2.68 (0.66)	−0.15 ± 2.53 (0.74)	0.92
Serum Creatinine (mg/dl)	Baseline	0.82 ± 0.15	0.86 ± 0.24	0.43
	60	0.83 ± 0.14	0.86 ± 0.22	—
	Mean diff (Baseline –Day 60) (*p* value)	0.01 ± 0.11 (0.61)	0.01 ± 0.08 (0.49)	1.0
Uric Acid (mg/dl)	Baseline	4.85 ± 1.60	4.86 ± 1.36	0.97
	60	4.60 ± 1.23	4.63 ± 1.33	—
	Mean diff (Baseline –Day 60) (*p* value)	−0.25 ± 0.80 (0.09)	−0.24 ± 0.57 (0.02)	0.95
Serum Sodium (mmol/L)	Baseline	141.35 ± 2.03	140.58 ± 3.82	0.32
	60	139.60 ± 6.84	140.19 ± 2.65	—
	Mean diff (Baseline –Day 60) (*p* value)	−1.75 ± 6.73 (0.15)	−0.39 ± 2.35 (0.36)	0.29
Serum Chloride (mmol/L)	Baseline	102.51 ± 2.16	101.63 ± 3.64	0.25
	60	102.77 ± 2.53	101.36 ± 3.16	—
	Mean diff (Baseline –Day 60) (*p* value)	0.26 ± 1.87 (0.44)	−0.27 ± 2.11 (0.48)	0.29

By Student “t” Test *p* > 0.05 was considered not significant.

**TABLE 13:** Comparison of mean laboratory data (Lipid profile) between groups.

Laboratory tests	Duration (Days)	Mean ( $\bar{Χ}$ ± SD)		*p* value
		Uthever (N = 31)	Placebo (N = 31)	
Total Cholesterol (mg/dl)	Baseline	178.55 ± 38.91	186.15 ± 50.70	0.51
	60	172.86 ± 31.89	179.79 ± 39.40	—
	Mean diff (Baseline –Day 60) (*p* value)	−5.68 ± 20.22 (0.12)	−6.36 ± 22.90 (0.13)	0.90
Serum Triglycerides (mg/dl)	Baseline	141.02 ± 77.48	168.46 ± 106.32	0.25
	60	132.48 ± 60.41	167.91 ± 198.89	—
	Mean diff (Baseline–Day 60) (*p* value)	−8.54 ± 61.85 (0.44)	−0.55 ± 127.61 (0.98)	0.75
LDL (mg/dl)	Baseline	115.51 ± 26.10	124.65 ± 42.13	0.30
	60	114.45 ± 30.75	119.42 ± 37.76	—
	Mean diff (Baseline –Day 60) (*p* value)	−1.06 ± 18.08 (0.74)	−5.23 ± 19.92 (0.15)	0.39

By Student “t” Test *p* > 0.05 was considered not significant.

## Discussion

The primary efficacy objective for the study was to find an anti-aging effect of Uthever. NAD^+^ was chosen as primary end point to demonstrate work out enhancing and anti-aging effect of Uthever supplement. NAD^+^ is an active component in the energy mechanism of the cells. Higher levels of NAD^+^ in the cells have been corelated to higher energy levels. NAD^+^ helps to produce ATP, which is a readily usable form of energy for the cells.

The other functions of NAD^+^ include protecting and repairing DNA, reducing DNA mutations, lowering cholesterol and strengthening the immune system, which could be responsible for the anti-aging effect of Uthever (Navas and Carnero, 2021).

Hence, the rise in the levels of NAD^+^/NADH at day 30 and day 60 illustrated the potential of Uthever to raise the levels of NAD^+^ in the cells. The rise in NAD^+^/NADH in active group was considerably higher than in the placebo, to have more significance, the dose or the duration could be increased further.

The second primary endpoint of the study was the 6 min walking endurance test. It was predicted that Uthever would improve the energy levels and thereby increase the walking capacity of the subjects. From the data, it was observed that the walking endurance of the subjects in the active i.e., Uthever group had a noteworthy rise at the end of the study.

To check the anti-aging effect of Uthever (NMN), the reduction of systolic blood pressure, diastolic blood pressure, and pulse pressure towards normalisation was checked at baseline, day 30, and day 60. There was no meaningful fall or rise towards normalization in the active or placebo group for diastolic or systolic blood pressure. This can be attributed to the comparatively lower dose of Uthever and the short duration of the study.

The final efficacy parameter was the SF-36 questionnaire score. The higher the score of SF 36, the better the wellbeing of the subject. There was a noteworthy rise in the SF 36 score seen in the Uthever group, which meant that the well-being of the subjects was increased by consuming Uthever for 60 days.

HOMA has been a chosen parameter to check the insulin sensitivity of the cells. With an increase in biological age, this capacity is decreased. Hence, increased sensitivity to insulin has been linked to anti-aging. For the same reason, the HOMA IR index was assessed as an exploratory objective in this study.

The analysis showed that there was no noteworthy change in HOMA score in the Uthever group, whereas there was a rise in the placebo group. Although the rise in the placebo group was marginal, and there was no statistical significance, we may co-relate this finding with the anti-aging effect of Uthever, as in its absence, the parameters worsened.

The results found in our study can be correlated with a few other clinical trials (Dollerup et al., 2018; Gale, 2004). NMN supplementation (250 mg/day) increased skeletal muscle insulin signalling, insulin sensitivity, and muscle remodelling in overweight or obese postmenopausal women. However, the effect of NMN was specific to insulin sensitivity in muscle and did not affect other important variables associated with insulin resistance, including indices of liver and adipose tissue insulin sensitivity, fasting plasma glucose, insulin, and adiponectin concentrations. Body composition (fat mass, fat-free mass, intra-abdominal adipose tissue volume, and intrahepatic triglyceride content), blood pressure, plasma glucose, insulin, free fatty acid, lipids, adiponectin and leptin concentrations, and both basal glucose and fatty acid kinetics did not change either (Yoshino et al., 2021). It is important to note that our study did not include particularly obese subjects, although there were no dietary restrictions imposed. The subjects were kept on their regular diet, and no improvements in the diets of the subjects were allowed during the trial.

The secondary objective of the study was to evaluate the safety of Uthever for the study duration. This was achieved through analysing the evaluation of adverse events and evaluation of deranged laboratory parameters and organ function tests like the liver function test (LFT) and renal function test (RFT).

In this study, the safety and efficacy of the oral administration of NMN-300 mg in healthy individuals was well established. The oral administration of NMN (300 mg) did not cause any specific deleterious effects in healthy volunteers, whereas nicotinamide has been known to adversely affect organs like kidneys, livers, and pancreatic beta cells and to induce nausea and headaches at higher doses, thus causing difficulty in using nicotinamide at high amounts to increase cellular NAD^+^. In this study, we did not observe adverse events such as nausea and hepatoxicity, as NMN did not increase nicotinamide blood levels enough to cause any such adverse events in this study. When we compared HOMA scores between groups, there was some indicative evidence that NMN supported the subjects’ ability to regulate their glucose levels to an extent, but this has to be looked at under various conditions such as increased dose vs. duration vs. diet regularities etc. Overall, NMN was well tolerated up to 300 mg.

The analysis of the data obtained in this study did not find any statistically significant changes in efficacy outcomes between the active and placebo groups. However, the increase in NAD^+^/NADH levels in serum, the improvement in overall health and walking endurance, are clinically significant. The initial results from this study seem to be signalling in the positive direction, but further large-scale trials with an increase in dose and duration of treatment are required to establish distinctive conclusions with statistical significance. This study opens up whole new horizons for trying NMN in areas like aging reversal, specific organ regeneration based on anti-aging and age-reversing effects, reversal of metabolic disorders, and even NMN’s effect on cancer prevention (Knip et al., 2000; Hwang and Song, 2020; Igarashi et al., 2021).

## Data Availability

The raw data supporting the conclusion of this article will be made available by the authors without undue reservation.
